# Supplementary material for: Milk Fat Globule Membrane Relieves Fatigue via Regulation of Oxidative Stress and Gut Microbiota in BALB/c Mice
Source: Antioxidants (Basel). 2023 Mar 13;12(3):712. doi: 10.3390/antiox12030712 (PMC10045747; doi:10.3390/antiox12030712)
Supplement: Supplementary file 1 [file antioxidants-12-00712-s001.zip › antioxidants-2145420-supplementary.pdf]

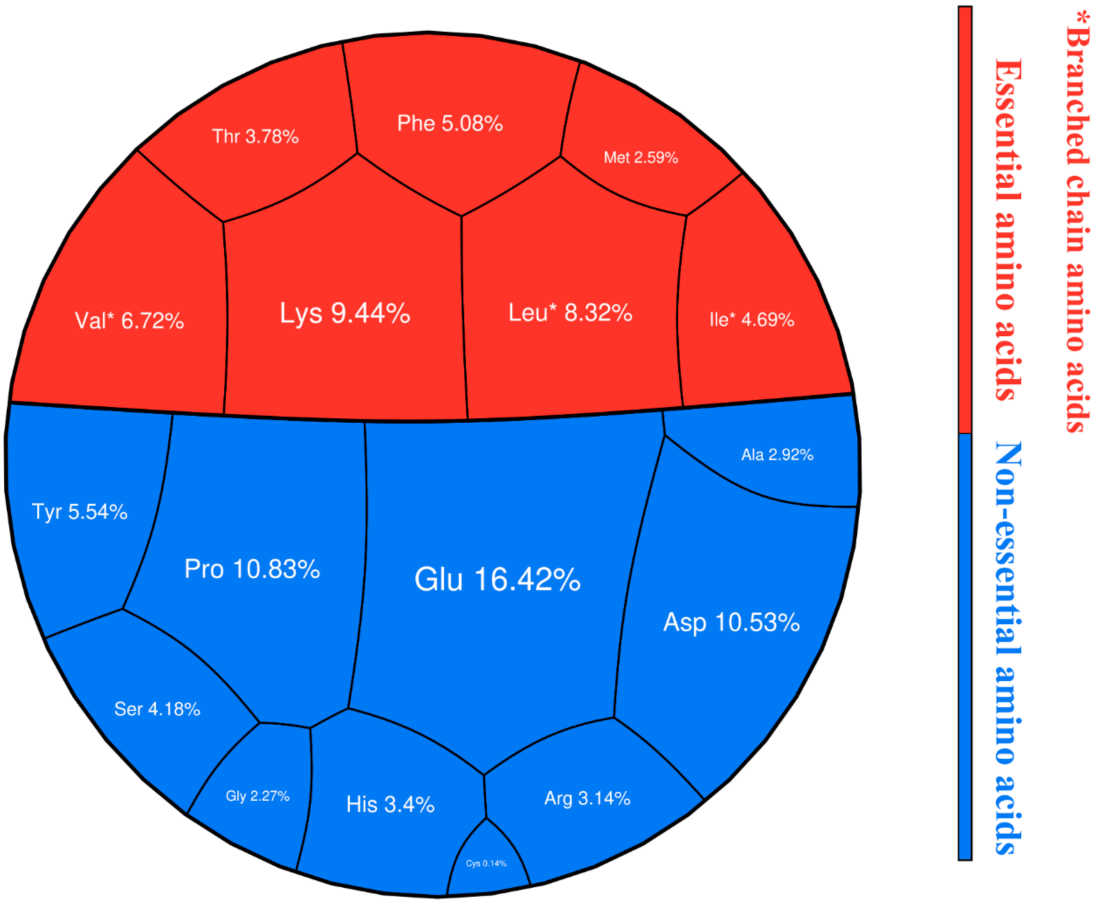

Figure S1. The amino acid composition of the milk fat globule membrane. Gly, Glycine; Ala, Alanine; Val, Valine; Leu, Leucine; Ile, Isoleucine; Met, Methionine; Phe, Phenylalanine; Trp, Tryptophan; Pro, Proline; Ser, Serine; Thr, Threonine; Cys, Cysteine; Tyr, Tyrosine; Asn, Asparagine; Gln, Glutamine; Asp, Aspartic acid; Glu, Glutamic acid; Lys, lysine; Arg, Arginine; His, Histidine. Data were shown as the percentage of each amino acid to the total amino acid.

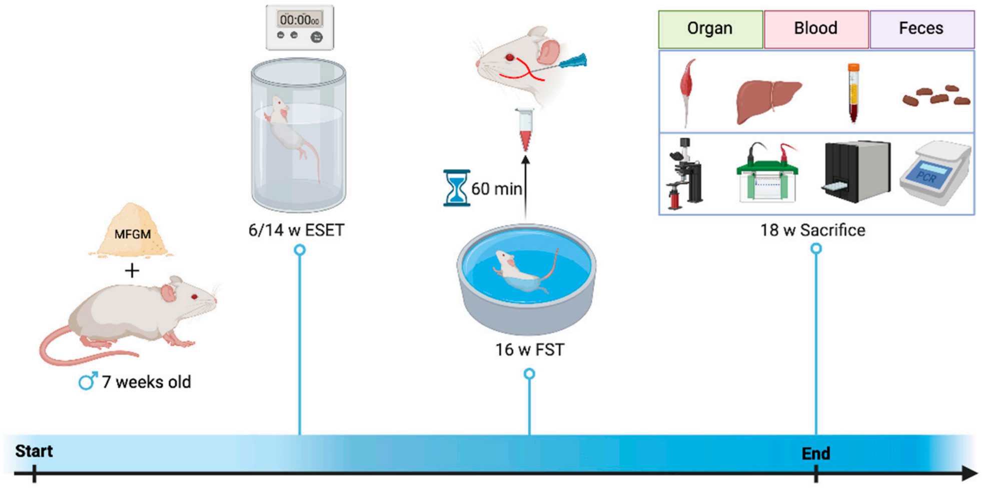

Figure S2. Detailed steps of the animal experiment. MFGM, milk fat globule membrane; ESET, exhaustive swimming exercise test; FST, free swimming test.
